# Age-associated neuronal micronuclei formation and transfer to microglia

**DOI:** 10.3389/fnagi.2026.1787252

**Published:** 2026-06-19

**Authors:** Chihiro Maeda, Yusuke Kishi, Ikuko Takeda, Masafumi Muratani, Hiroaki Wake, Fuminori Tsuruta

**Affiliations:** 1 Doctoral Program in Biology, Degree Programs in Life and Earth Sciences, Graduate School of Science and Technology, University of Tsukuba, Tsukuba, Ibaraki, Japan; 2 Institute for Quantitative Biosciences, The University of Tokyo, Bunkyo-ku, Tokyo, Japan; 3 Graduate School of Pharmaceutical Sciences, The University of Tokyo, Bunkyo-ku, Tokyo, Japan; 4 Division of Aging Biology, Research Institute for Science and Technology, Tokyo University of Science, Noda, Chiba, Japan; 5 Department of Anatomy and Molecular Cell Biology, Nagoya University Graduate School of Medicine, Nagoya, Aichi, Japan; 6 Division of Multicellular Circuit Dynamics, National Institute for Physiological Sciences, Okazaki, Aichi, Japan; 7 Department of Genome Biology, Institute of Medicine, University of Tsukuba, Tsukuba, Ibaraki, Japan; 8 Department of Physiological Sciences, Graduate University for Advanced Studies, SOKENDAI, Hayama, Japan; 9 Center of Optical Scattering Image Science, Department of Systems Science, Kobe University, Kobe, Japan; 10 Master’s and Doctoral Program in Biology, Institute of Life and Environmental Sciences, University of Tsukuba, Tsukuba, Ibaraki, Japan; 11 Ph.D. Program in Human Biology, School of Integrative and Global Majors, University of Tsukuba, Tsukuba, Ibaraki, Japan; 12 Ph.D. Program in Humanics, School of Integrative and Global Majors, University of Tsukuba, Tsukuba, Ibaraki, Japan; 13 Master’s and Doctoral Program in Neuroscience, Graduate School of Comprehensive Human Sciences, University of Tsukuba, Tsukuba, Ibaraki, Japan; 14 Center for Quantum and Information Life Sciences, University of Tsukuba, Tsukuba, Ibaraki, Japan

**Keywords:** extracellular matrix, microglia, micronuclei (MN), neuron, nuclear envelope

## Abstract

Microglia, the resident immune cells of the central nervous system, dynamically respond to signals from their microenvironment, including adjacent neurons. Among these signals, nuclear contents released from damaged neurons have been implicated in triggering inflammatory microglial responses. Recently, we found that micronuclei (MNs) derived from neurons during the early postnatal stage act as intercellular mediators that alter the microglial characteristics. However, it remains unclear whether a similar mechanism occurs in the aging brain. In this study, we report that neuronal MNs are formed and transferred to microglia during aging. The neuronal nuclear envelope became fragile with aging and forms MNs in association with nuclear envelope invagination. Subsequently, neuronal MNs were taken up by adjacent microglia. In contrast to the developmental stage, microglia incorporating MNs exhibited extended processes during the aged stage. We also identified several candidate genes whose expression patterns were altered in microglia following MN incorporation. These findings suggest that MN incorporation alters the characteristics and functions of microglia in the aging brain. Our data propose an unrecognized neuron-to-microglia communication in the aged brain mediated by MN propagation.

## Introduction

Aging is a multifaceted biological process characterized by chronic inflammation, a gradual decline in physiological function and behavioral capacity. Given that the brain serves as the central regulator of systemic physiology and is a highly complex organ, brain aging is driven by exceptionally intricate mechanisms (Tosato et al., 2007; Maeda and Tsuruta, 2024). Among the cellular components of the brain, glial cells, such as microglia and astrocytes, play pivotal roles in regulating cerebrovascular function, maintaining the central nervous system (CNS) interface, and mediating phagocytic clearance of waste products during aging (Prinz et al., 2021).

Microglia are resident immune cells in the CNS and modulate both neural and cerebrovascular networks (Csaszar et al., 2022). Microglia constitute a highly heterogeneous population throughout postnatal development and aging (Hammond et al., 2019; Li et al., 2023). This heterogeneity plays a crucial role in the multilayered regulation of their functions. It has been suggested that microglial alterations are influenced not only by intrinsic genetic programs but also by extracellular stimuli, including signals from neurons and astrocytes, as well as interactions with the extracellular matrix. Recently, we have reported that micronuclei (MNs) are transferred from neurons to microglia during prenatal development (Yano et al., 2025). MNs are small extranuclear structures formed due to chromosome segregation errors or physical stress encountered during cellular migration (Crasta et al., 2012; Denais et al., 2016; Raab et al., 2016). We found that MNs are generated as neurons undergo migratory stress while passing through narrow spaces. Importantly, microglia that internalize MNs exhibited significant alterations of extracellular matrix (ECM)-related gene expression, including *Col1a2, Col1a1,* and *Col3a1*. These findings suggest that MNs may serve as a novel intercellular signal between neurons and microglia during developmental stages. However, whether MNs derived from aged neurons exist in the brain under physiological conditions remains largely unexplored.

Recent studies have revealed that neuronal nuclei undergo profound functional and structural remodeling during aging. Age-associated alterations in nuclear architecture have emerged as a fundamental hallmark of brain aging (Lu et al., 2004; Das and Ramanan, 2023). Aged neurons exhibit nuclear abnormalities, including increased nuclear envelope fragility and stiffness, reduced expression of nuclear envelope proteins, and disrupted chromatin organization (Bedrosian et al., 2021; Zhang et al., 2022; Frey et al., 2023; Zhang et al., 2023). Supporting a causal link between nuclear architecture and aging, Hutchinson-Gilford progeria syndrome—a premature aging disorder—displays severe defects in nuclear envelope integrity driven by mutations in Lamin A/C (Goldman et al., 2004; Scaffidi and Misteli, 2006). In addition, neurodegenerative disorders, such as amyotrophic lateral sclerosis and Alzheimer’s disease, show pronounced abnormalities in nuclear envelope morphology, such as membrane infoldings and invaginations (Paonessa et al., 2019; Chen et al., 2020). Collectively, these observations suggest that alterations in nuclear morphology and nuclear envelope components are central features of neuronal aging and age-related neurodegeneration.

Here, we report that MNs are formed in neurons under physiological aging conditions. We found that neuronal MNs are generated in association with nuclear envelope invagination. The number of MNs formation occurred in the deep layers of the cerebral cortex. In contrast to the postnatal stage, aging-associated micronuclei frequently contained little chromatin or consisted predominantly of nuclear membrane fragments. Following their formation in neurons, these MNs are taken by microglia, accompanied by an alternation in microglial morphology. Bulk RNA-seq analysis revealed that MN-positive (MN^+^) microglia exhibit upregulated expression of a heparan sulfate (HS)–related gene cascade. Together, these findings suggest that neuronal MNs contribute to the acquisition of distinct functional states in aged microglia.

## Methods

### Mice

The C57BL/6 J aged mice (19–25 months of age) and young mice (19–25 months of age) were used in this this study. Aged mice were obtained from National BioResource Project, and young mice were obtained from Japan SLC, Inc. SLC. To generate NexCre: LSL-Sun1-GFP mice, the LSL-Sun1-GFP mice were crossed with B6; 129-Gt (ROSA) 26Sor^tm5(CAG-Sun1/sfGFP)Nat/J^ (LSL-Sun1-GFP) (JAX strain# 021039) and Neurod6^tm1(cre)Kan^ (NexCre) (Goebbels et al., 2006; Mo et al., 2015). Mice were housed in a 12-h light (light on at 8:00 a.m.)/12-h dark cycle (light off at 8:00 p.m.) at constant room temperature and humidity. All animal experiments were conducted according to the university guidelines for animal care. This study was approved by the Animal Experiment Committee at the University of Tsukuba (the approval numbers: 23–370, 24–377, 25–365).

### Antibodies

Rabbit anti-GFP pAb (#598, MBL, 1:500), Chicken anti-GFP pAb (ab13970, abcam, 1:1000), Rabbit anti-*β*-tubulin pAb (MMS-435P, BioLegend, 1:50), Rabbit anti-MAP2 mAb (MAB378, Merck Millipore, 1:500), Goat anti-Iba1 pAb (ab5076, abcam, 1:200), Rat anti-GFAP mAb (G3893, SIGMA, 1:500), and Mouse anti-DNA mAb (AC-30-10, Merck, 1:50) were used as the primary antibodies for immunofluorescence. Donkey anti-chicken IgY (H + L) Alexa Fluor 488 (A78948, Invitrogen, 1:1000), Donkey anti-rabbit IgG (H + L) Alexa Fluor 488 (ab150073, abcam, 1:1000), Donkey anti-mouse IgG (H + L) Alexa Fluor 594(ab150108, abcam, 1:1000), Donkey anti-rabbit IgG (H + L) Alexa Fluor 594 (ab150076, abcam, 1:1000), Donkey anti-goat IgG (H&L) Alexa Fluor 594 (ab150132, abcam, 1:1000), Donkey anti-rat IgG (H&L) Alexa Fluor 647 (ab150155, abcam, 1:500), Donkey anti-rabbit IgG (H&L) Alexa Fluor 647 (ab150075, abcam, 1:500) were used as the secondary antibody. The anti-CD11b conjugated with APC-Cy7 (BioLegend,cat. no. 101226, 1:400) was used for FACS analysis.

### Immunohistochemical staining

For assessment of pharmacological inhibition of microglial phagocytosis, mice were used and randomly assigned to a vehicle group or an MRS2578-treated group. MRS2578 was first dissolved in dimethyl sulfoxide (DMSO) and then diluted with sterile PBS immediately before administration. MRS2578 was administered intraperitoneally at a dose of 3 mg/kg once daily for three consecutive days, and were then anesthetized by isoflurane inhalation (Pfizer). After perfusion with PBS, brains were removed and fixed by perfusion with 4% paraformaldehyde (PFA) in PBS. Brains were then cryoprotected in 30% sucrose in PBS, washed with PBS, embedded in O.C.T. compound (Sakura Fine Tech Japan) on dry ice, and stored at −30 °C. Coronal sections (50 μm) were prepared using a cryostat (CM1950, Leica) set to −25 °C. Sections were stored in cryoprotectant (50% glycerol in PBS) at −30 °C. Free-floating sections were permeabilized and blocked in PBS containing 0.25% Triton X-100 and 5% bovine serum albumin (BSA). Sections were incubated with primary antibodies with Can Get Signal Solution B (NKB-601, TOYOBO) at 4 °C for 2 days. Sections were washed three times in PBS (10 min each). Secondary antibody incubation was performed with Can Get Signal Solution B at room temperature for 3 h, followed by secondary antibody detection. Sections were counterstained with DAPI (Dojindo, 1:1000) and mounted with VECTASHIELD Mounting Medium (Vector Laboratories). Images were acquired using a confocal laser scanning microscope (LSM700, Carl Zeiss) with 40 × (Plan-Apochromat 40×/0.8 M27) and 63 × (Plan-Apochromat 63×/1.3 Oil DIC M27) objective lenses. Z-stack images were acquired at 1 μm intervals using ZEN software (LSM700; ZEN 2009, Carl Zeiss).

### Transmission electron microscopy

For electron microscopy, brains from 24-month-old C57BL/6 J mice were perfused with fixative (0.1 M phosphate buffer pH 7.2, containing 4% PFA, 2.5% glutaraldehyde, 0.1% picric acid, and 0.05 mg/mL ruthenium red). Brains were post-fixed in the same fixative at 4 °C for 1 day. After washing, brain sections were prepared and post-fixed with 1% osmium tetroxide at room temperature for 2 h. Samples were dehydrated through graded ethanol and exchanged into propylene oxide. Ultrathin sections (60–80 nm) were prepared using an UltraCut UCT (Leica) and stained with 2% uranyl acetate and lead citrate. Images were acquired using a JEM 1400 (JEOL).

### Two-photon microscope *in vivo* imaging

The two-photon in vivo imaging was performed using 18-month-old NexCre: LSL-Sun1-GFP mice (Haruwaka et al., 2019; Takeda et al., 2022; Yano et al., 2025). Mice were initially anesthetized with a ketamine–xylazine combination. Following skull exposure, a custom-designed head plate was secured using dental cement (G-CEM ONE, GC). A craniotomy of 2 mm diameter was made, and a glass window composed of two coverslips (2 and 3.5 mm, Matsunami Glass) and an ultraviolet curable adhesive (NOR-61, Norland Products, Jamesburg, NJ) was placed over the cortical surface and fixed with UV resin (UV craft resin, KIYOHARA, Osaka, Japan). The perimeter of the cranial window was reinforced with a mixture of dental cement and adhesive resin cement (Super Bond, Sun Medical). Imaging of the cerebral cortex was conducted with a two-photon laser scanning system (AX R MP; Nikon Instech Co., Tokyo, Japan) equipped with a water-immersion objective lens (Apo Apo LWD 20x/1.0w; Nikon) and a Ti:sapphire laser (InSight X3+, Spectra-Physics, Milpitas, CA) tuned to 920 nm. Z-stack time-lapse images were collected at 1 μm intervals every minute using NIS-Elements AR 6.20.00 (Build 2057). FIJI ImageJ was employed to convert the Z-stacks (50 μm total images, 1 μm each interval) into single projection images using average intensity projection.

### Image analysis

For MN formation analysis, Z-stack data acquired with ZEN were converted into a single TIFF file using FIJI ImageJ (version 2.16.0/1.54p). The TIFF data were imported into the MATLAB-based program CAMDi (Yano et al., 2021). Imported data were binarized, and MN number, area, and volume were quantified. For the position of MN formation analysis, angles between nuclear envelope invagination sites and MN formation sites were quantified using the ImageJ angle measurement tool. The neuronal nucleus was outlined using the Ellipse Selection Tool. In *Analyze* > *Set Measurements*, the Centroid option was selected and *Analyze* > *Measure* was used to obtain nucleus center coordinates (X, Y) from the Results window. Using the angle tool, three points were selected in order: the nuclear invagination point, the nuclear center point, and the MN formation point. The resulting angles were recorded from the Results window. Microglial morphology was analyzed using ImageJ skeleton analysis. Confocal images (LSM700, Carl Zeiss) were processed by maximum intensity projection (MIP) of 3D stacks using the Iba1 signal. MIP images were binarized, noise was removed, and images were skeletonized (*Process > Binary > Skeletonize*). Quantification was performed using *Plugins > Skeleton > Skeletonize (2D/3D)*. Endpoints were used as a parameter reflecting the branch number.

### Fluorescence-activated cell sorting

To isolate GFP^−^ and GFP^+^ microglia, all processes were carried out on ice, and all reagents were kept cold to reduce artificial activation of microglia. The cerebral cortex was collected from 22-months-old of NexCre: LSL-Sun1-GFP mice and homogenized in 500 μL PBS containing 0.2% DNase I (TAKARA). Homoginization was conducted stepwise using 23G and 27G needles. The homogenate was combined with 145 μL Debris Removal Solution (Miltenyi). 600 μL of PBS was layered on top, followed by centrifugation at 3,000 ×*g* for 10 min at 4 °C. After removing the supernatant, samples were resuspended in 600 μL of PBS and centrifuge again at 1,000 ×*g* for 10 min at 4 °C. The cells pellets were resuspended in 200 μL PBS containing 0.2% BSA, stained with anti-CD11b APC-Cy7 (BioLegend, #101226), and incubated on ice for 10 min. Samples were mixed with 60 μL Debris Removal Solution and overlaid with 260 μL PBS. After centrifugation and removal of the supernatant, pellets were resuspended in 200 μL of PBS containing 0.2% BSA. GFP^−^ and GFP^+^ microglia were then sorted using a FACS Melody (Becton Dickinson).

### RNA-seq analysis

RNA-seq libraries were constructed from 10,000 FACS-isolated cells. Total RNA was extracted using ISOGEN II reagent (Nippon Gene, Tokyo, Japan). The RNA-seq analysis procedure was conducted according to a previous study (Husna et al., 2024). Briefly, each sequencing library preparation used 3.5 μL of purified RNA solution using SMART-seq Stranded Kit (Takara Bio, cat. no. 634443). Size distribution and concentration of amplified cDNA libraries were validated by an Agilent Bioanalyzer (Agilent, CA, USA) and an Agilent High-Sensitivity DNA kit (Agilent). Sequencing was conducted with a NextSeq500 sequencer (Illumina, CA, USA) following the manufacturer’s instructions. Reads in FASTQ files were imported into CLC Genomics Workbench (CLC-GW, ver.10.1.1, Qiagen), mapped to mouse (mm10) reference genome, and quantified using a 49,585-gene annotation set (downloaded from the CLC-GW server) to obtain the total count values, which were combined into a table.

### Omics data analysis

For visualization of RNA-seq data, we focused on genes related to HS. HS-related genes were defined based on the literature as genes involved in HS chain biosynthesis, modification, degradation, and core proteoglycans. From the normalized gene expression matrix, only the predefined HS-related genes were extracted for analysis (Bishop et al., 2007; Iozzo et al., 2009). To generate the heatmap, expression values from all MN^−^ and MN^+^ microglia samples were used, and gene-wise Z-score normalization (mean = 0, standard deviation = 1) was applied to each gene. This normalization removed differences in absolute expression levels between genes and enabled comparison of relative expression patterns across samples. Hierarchical clustering was performed based on the similarity of gene expression patterns using correlation distance, and gene order was determined by average linkage clustering. The final heatmap displays HS-related genes as rows and MN^−^ and MN^+^ microglia samples as columns, with colors representing Z-score–normalized expression values. For the pathway enrichment analysis, differentially expressed genes (DEGs) between MN − and MN + microglia were defined as genes satisfying FDR < 0.05 and |log2 fold change| ≥ 2, calculated from normalized RNA-seq expression values. Genes upregulated in MN + or MN − microglia were analyzed separately for pathway enrichment using Enrichr through the gseapy Python package. Enrichment analysis was performed across multiple gene set libraries, including GO Biological Process 2021, GO Molecular Function 2021, GO Cellular Component 2021, Reactome 2022, and KEGG 2019 Mouse. The enrichment results were visualized using dot plots. GeneRatio represents the proportion of genes overlapping with each pathway, and the color scale indicates −log10 (FDR). For each group, the top 12 pathways ranked by adjusted *p*-value were displayed. To compare gene expression between MN^−^ and MN^+^ microglia, a scatter plot was generated using the mean normalized expression values for each group. Tabular data containing gene names, mean normalized expression values for MN^−^ and MN^+^ microglia, and FDR values were imported using Python (pandas). For each gene, log2 fold change was calculated as log2(MN^+^/ MN^−^). In the scatter plot, the x-axis represents the mean normalized expression in MN^−^ microglia and the y-axis represents that in MN^+^ microglia. To improve the visualization of lowly expressed genes, log1p transformation was applied to both axes. Differentially expressed genes were defined as those satisfying FDR < 0.05 and |log2FC| ≥ 1, and were classified into three groups: MN^+^ upregulated, MN^−^ upregulated, and not significant, which were displayed using different colors. To highlight genes with prominent differences, a score was calculated as the product of -log10 (FDR) and |log2FC|. Based on this score, the top 10 MN^+^ upregulated genes and the top 10 MN^−^ upregulated genes (20 genes in total) were selected and annotated in the scatter plot. Figures were generated and saved using Matplotlib. Genes with a fold change ≥5 in GFP^+^ cells relative to GFP^−^ cells were defined as upregulated. Functional enrichment analysis was performed using Enrichr^1^.

### Statistics and reproducibility

Statistical analyses were performed using GraphPad Prism version 10.4.1 (GraphPad Software). Comparisons between two groups were performed using Student’s *t*-test. For comparison among the multiple group comparison, the one-way analysis of variance (ANOVA) Tukey’s multiple comparison test was used.

## Results

### Preferential formation of MN in deep cortical layers of the aged brain

**Figure 1:**
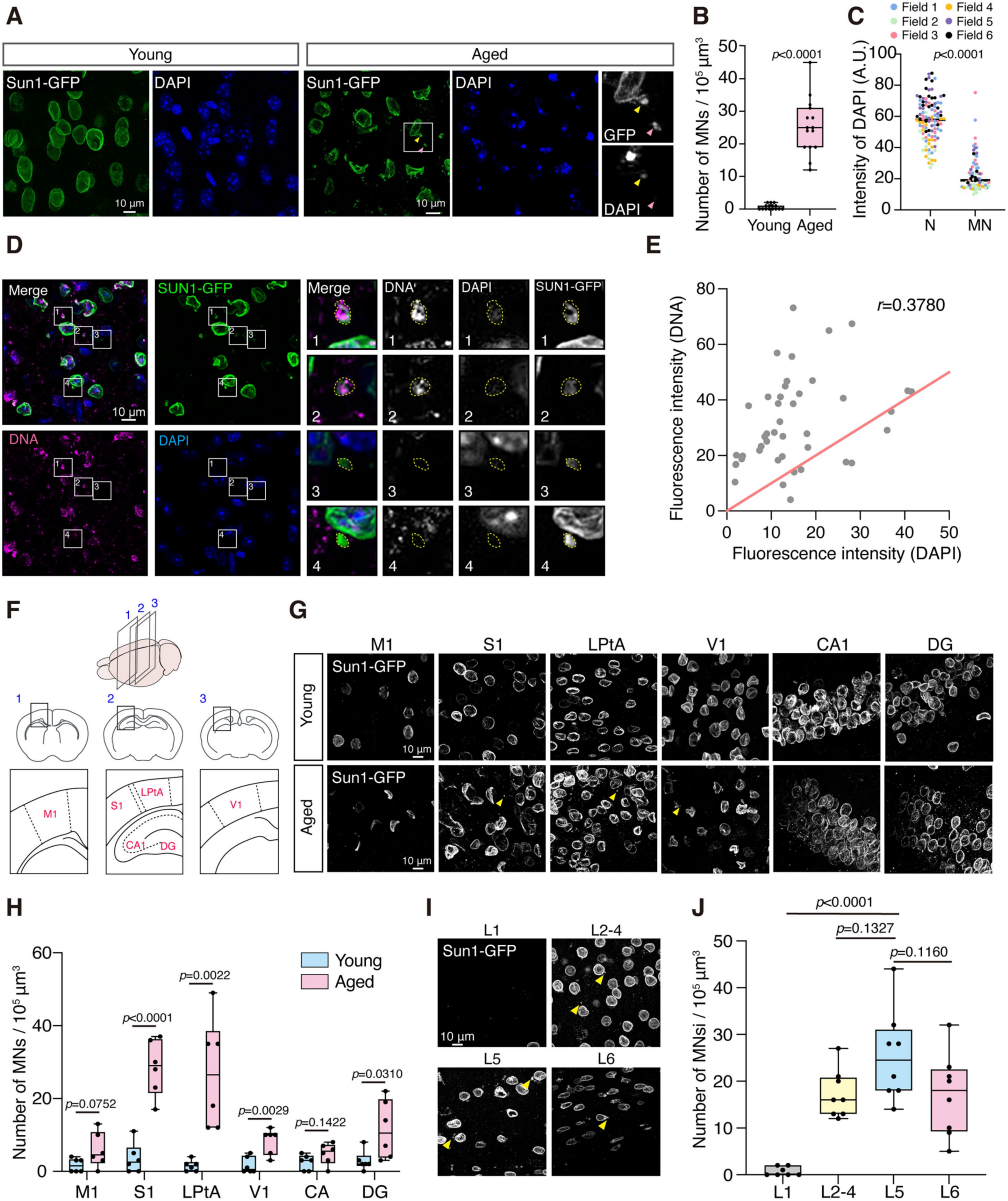
Preferential formation of MN in deep cortical layers of the aged brain. **(A)** Immunostaining of SUN1-GFP in the cerebral cortex of young mouse (2 months of age) and aged mouse (20 months of age). Yellow arrowhead indicates DAPI^+^ MN, pink arrowhead indicates DAPI^−^ MN. **(B)** Quantification of the number of MNs. Fifteen fields obtained from 3 brains, young mice; 1, 2, and 2 months of age, aged mice; 20, 21, and 25 months of age, mean ± SEM, *p* values were analyzed by Student *t*-test. **(C)** Quantification of the fluorescence intensity of nuclei and micronuclei. Data were obtained from 6 fields derived from the same 3 mice used in **(A,B)**. Each color represents measurements from an individual imaging field, mean ± SEM, *p* values were analyzed by Student *t*-test. **(D)** Immunostaining of DNA and SUN1-GFP in the cerebral cortex of aged mouse (22 months of age). Yellow dot line indicates the area of MN. **(E)** Scatter plot of DAPI and DNA signal intensities, 43 MNs obtained from 3 brains, 19, 22, 23 months of age, Spearman’s rank correlation coefficient test. **(F)** Schematic illustration of the brain area in **(E,F)**. **(G)** Immunostaining of SUN1-GFP in the cerebral cortex of young mouse (2 months of age) and aged mouse (20 months of age). Arrowhead indicates MN. M1, primary motor cortex; S1, primary somatosensory cortex; LPtA, lateral Posterior association cortex; V1, primary visual cortex; CA1, Cornu Ammonis 1; DG, dentate gyrus **(H)** Quantification of the number of MNs. Six fields obtained from 3 brains, young mice; 1, 2, and 2 months of ages, aged mice; 20, 21, and 25 months of age. The number of primary nuclei per field; M1 (young: 23 ± 8.3, aged: 31 ± 13), S1 (young: 39 ± 16, aged: 31 ± 13), LtPa (young: 34 ± 15, aged: 37 ± 15), V1 (young: 55 ± 23, aged: 43 ± 18), CA (young: 65 ± 27, aged: 38 ± 3.0), and DG (young: 50 ± 5.6, aged: 23 ± 3.1), mean ± SEM, *p* values were analyzed by Student *t*-test. **(I)** Immunostaining of nuclear envelope in the cerebral cortex of aged mouse (19 months of age). Arrowhead indicates MNs. **(J)** Quantification of the number of MNs. Eight fields obtained from 2 brains, aged mice: 19 months of age. The number of primary nuclei per field; L1: 0.13 ± 0.13, L2-L4: 29 ± 1.9, L5: 14 ± 0.67, and L6: 25 ± 2.2, mean ± SEM, *p* values were analyzed by one-way analysis of variance (ANOVA) Tukey’s multiple comparisons test.

We previously reported that MNs are generated in neurons during early postnatal development. To examine whether MNs are also present in the aged brain, we analyzed the cerebral cortex using NexCre; LSL-SUN1-sfGFP-Myc mice (NexCre; LSL-Sun1-GFP), which enable specific labeling of the nuclear envelope of excitatory neurons (Goebbels et al., 2006; Mo et al., 2015). We found a significant increase in the number of MNs in aged neurons compared with young neurons (Figures 1A,B). Notably, aged brain micronuclei showed lower DNA content (Figure 1C). As immunofluorescence of DAPI is not sensitive (Arvanitaki et al., 2024), we stained MNs using anti-DNA antibody. DNA staining appeared to be partially more sensitive than DAPI staining; however, only a weak correlation was observed between DAPI and DNA staining (Figures 1D,E). These results suggest that micronuclei in the aged brain contain low amounts of DNA or may even lack DNA. MNs were primarily detected in the cerebral cortex, with pronounced enrichment in the somatosensory and lateral posterior association cortex, and were absent in the visual cortex, motor cortex, and hippocampus (Figures 1F–H). In addition, MN production is not dependent on the density of primary nuclei. Within MN-enriched cortical regions (somatosensory cortex and lateral posterior association cortex), MNs were preferentially localized to deep cortical layers of the aged brain, particularly layers 5 and 6 (Figures 1I,J). Taken together, these data suggest that aged cortical neurons form MNs at a higher frequency than young neurons.

### Abnormalities in the neuronal nuclear envelope are associated with MN formation

**Figure 2:**
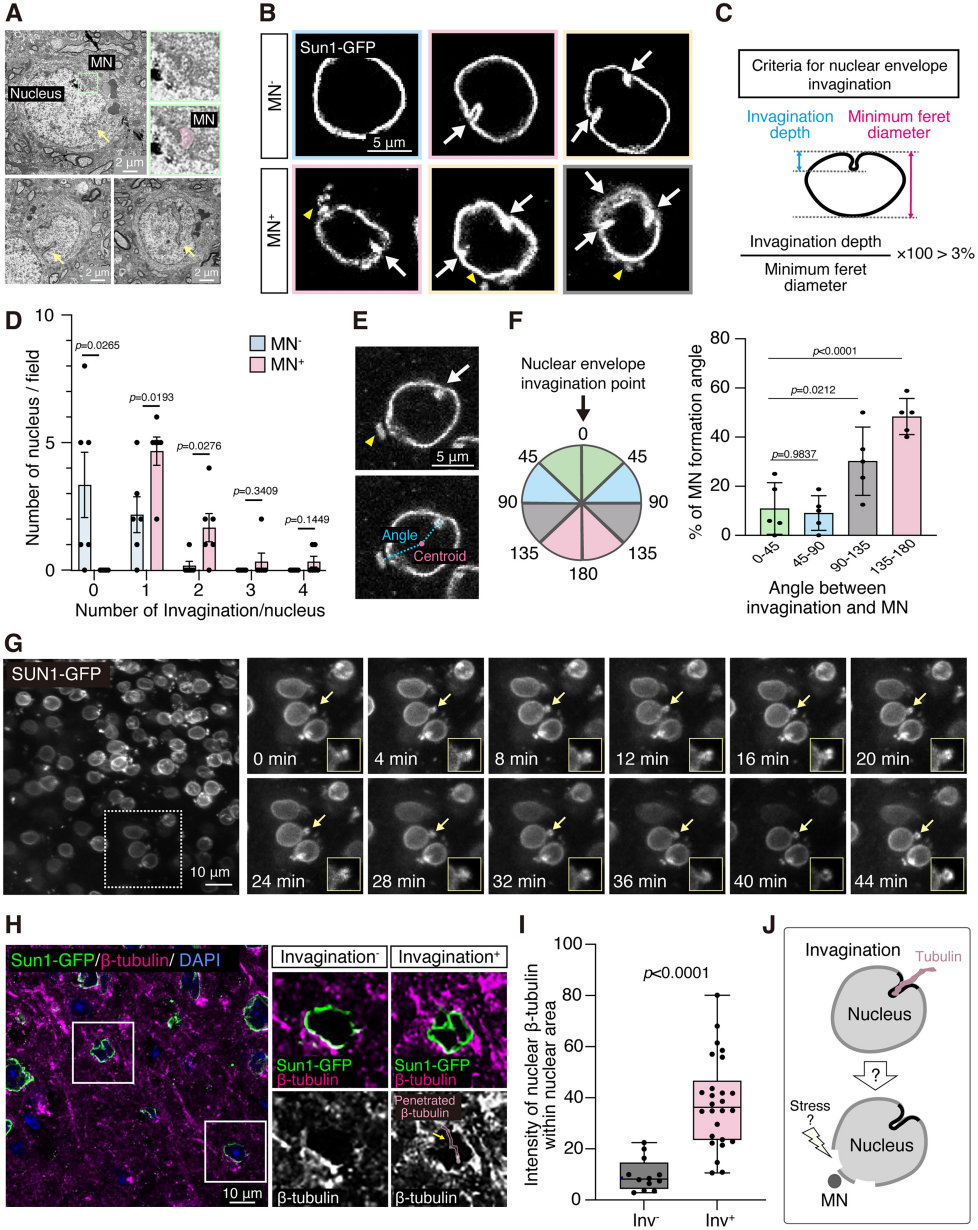
Preferential formation of MN in deep cortical layers of the aged brain. **(A)** TEM images of the cerebral cortex of aged mice (25 months of age). Magenta; MN. Yellow arrows indicate the invagination. **(B)** Immunostaining of SUN1-GFP in the cerebral cortex of aged mouse (19 months of age). Arrowhead indicates the MNs, Arrow indicates nuclear envelope invagination site. **(C)** Schematic illustration of the definition of nuclear envelope invagination. **(D)** Quantification of the number of nuclear envelope invagination with or without MNs. Six fields obtained from 3 brain, 19, 19 and 20 months of age, MN^−^; 34 nuclei, MN^+^; 42 nuclei. Mean ± SEM, *p* values were analyzed by Student *t*-test. **(E)** Immunostaining of nuclear envelope in the cerebral cortex of aged mouse (19 months of age). Arrowhead indicates the MNs, Arrow indicates nuclear envelope invagination site. **(F)** Pie chart shows the illustration of the relationship between invagination site and MN formation site (left). Quantification of the angle between invagination site and MN formation site (right). Five fields, 19 months of age, *n* = 87. *p* values were analyzed by one-way ANOVA Dunnett’s multiple comparisons test. **(G)** Two-photon *in vivo* imaging of the SUN-GFP mice (18 months of age). The interval of taking images was every 1 min (Supplementary Video S1). The arrows indicate MN. **(H)** Immunostaining of SUN1-GFP and *β*-tubulin in the cerebral cortex of aged mouse (20 months of age). Arrow indicates the penetrated β-tubulin. **(I)** Quantification of the fluorescence intensity of β-tubulin in the nucleus with or without invaginations. Invagination-negative (Inv^−^); 12 nuclei, Invagination-positive (Inv^+^); 26 nuclei. Eight fields obtained from 3 brains (20, 21, and 25 months), mean ± SEM, *p* values were analyzed by Student *t*-test. **(J)** Working hypothesis of the MN formation following nuclear envelope invagination. Mechanical stress on the side opposite the nuclear envelope invagination induces changes in the nuclear envelope and adjacent chromatin architecture, thereby promoting the formation of MNs.

Next, we investigated the mechanisms underlying MN formation in aged neurons. Using transmission electron microscopy (TEM), we observed prominent nuclear envelope invaginations in the nuclei of aged neurons undergoing MN formation (Figure 2A). To assess the relationship between MN formation and nuclear envelope invagination, we quantified the number of invaginations in nuclei with or without MNs. More than half of MN^−^ nuclei exhibited no detectable nuclear envelope invaginations. In contrast, the majority of MN^+^ nuclei displayed one to three nuclear envelope invaginations per nucleus (Figures 2B–D). We next assessed the spatial relationship between nuclear invagination and MN formation by measuring the angles defined by the neuronal center, the invagination site, and the MN formation site. Approximately half of MNs formed during aging were positioned on the side opposite to the nuclear invagination site (Figures 2E,F). To further investigate this phenomenon, we performed *in vivo* imaging using two-photon excitation microscopy and were able to observe micronuclei formation (Figure 2G). Based on this spatial observation, we sought to identify factors involved in nuclear invagination associated with MN formation. Nuclear invaginations have previously been reported in neurons from patients with neurodegenerative diseases and have been linked to aberrant microtubule localization (Paonessa et al., 2019; Chen et al., 2020). Consistent with this, we detected *β*-tubulin within the nucleus, where it was preferentially enriched at nuclear envelope invagination sites. Quantitative analysis revealed a significant increase in intranuclear β-tubulin levels in nuclei exhibiting nuclear envelope invagination (Figures 2H,I). Together, these results suggest that nuclear envelope invagination in physiologically aged neurons is associated with cytoskeletal abnormalities and is closely linked to MN formation during neuronal aging (Figure 2J).

### Neuronal MN propagates to microglia in the aged brain

**Figure 3:**
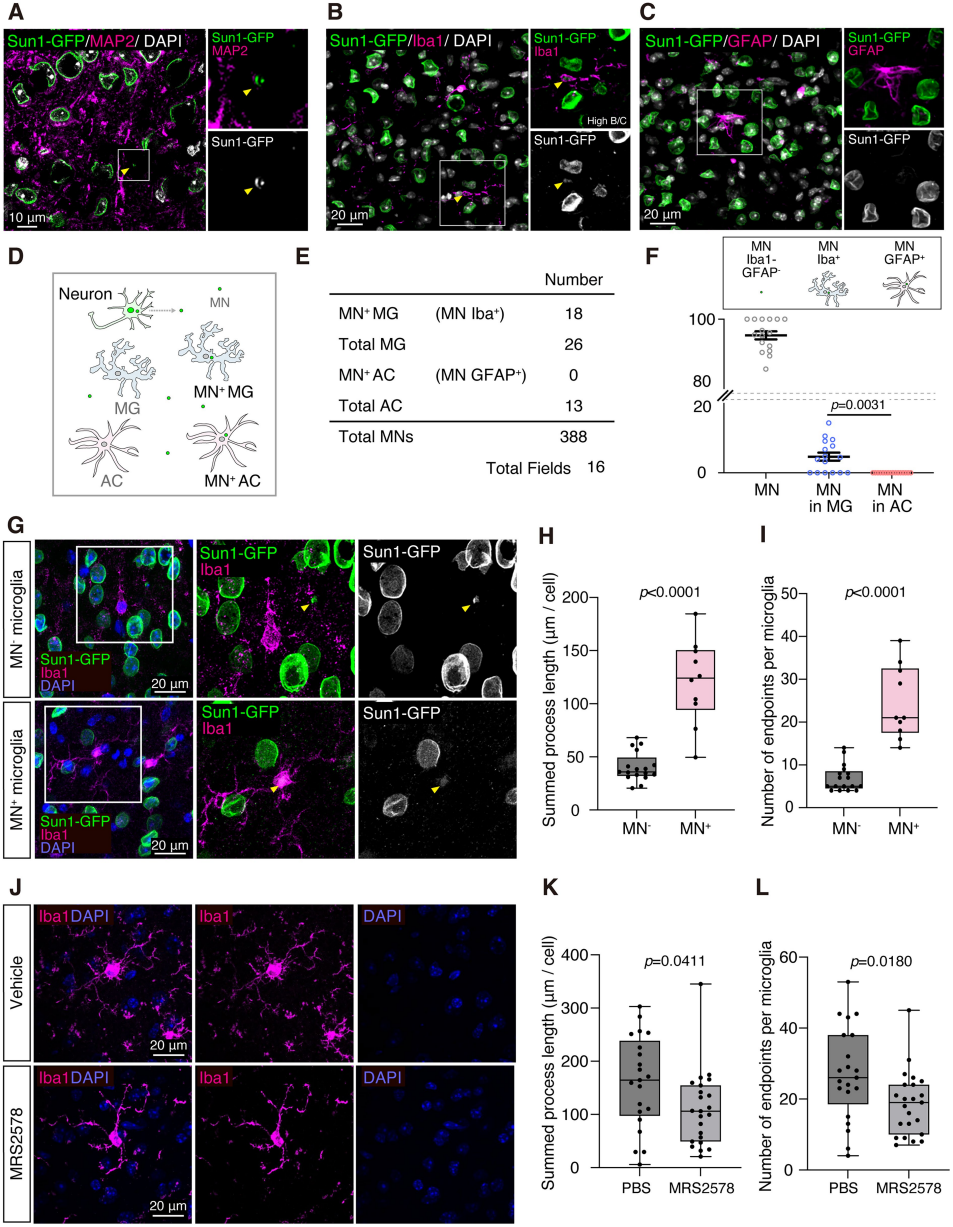
Neuronal MN propagates to microglia in the aged brain. **(A)** Immunostaining of SUN1-GFP with MAP2 in the cerebral cortex of aged mouse (19 months of age). Arrowhead indicates MNs. **(B)** Immunostaining of SUN1-GFP with Iba1 in the cerebral cortex of aged mouse (25 months of age). Arrowhead indicates MNs. High B/C, increasing brightness and contrast. **(C)** Immunostaining of SUN1-GFP with GFAP in the cerebral cortex of aged mouse (25 months of age). **(D)** Illustration of extracellular MN (MN), MNs^+^ neurons, MNs^+^ astrocytes (MN^+^ AC), and MNs^+^ microglia (MNs^+^ MG). MG; microglia, AC astrocytes. **(E,F)** Quantification of the proportion of MNs^+^, MN: extracellular MNs, MN in AC: MNs taken by AC, MN in MG: MNs taken by MG. Sixteen fields, 3 mice brain (20, 20, and 25 months of age). mean ± SEM, *p* values were analyzed by one-way analysis of variance (ANOVA) Tukey’s multiple comparisons test. **(G)** Immunostaining of Iba1^+^ microglia with or without MN, 20 months of age. Arrowhead indicates the MNs. **(H,I)** Graph showing process length **(H)** and endpoints **(I)** in the presence or absence of MNs. Twenty-seven fields obtained from 3 brains (19, 21, and 25 months of age) MN^−^; *n* = 17, MN^+^; *n* = 10. mean ± SEM, *p* values were analyzed by Student *t*-test. **(J)** Immunostaining of Iba1^+^ microglia in the cerebral cortex of aged mice (30 months of age) treated with PBS or MRS2578. **(K,L)** Graph showing process length **(H)** and endpoints **(I)** with or without MRS2578 administration. Forty-four fields obtained from 3 mice brains, 30 months of age, vehicle; *n* = 3, MRS2578; *n* = 3. Mean ± SEM, *p* values were analyzed by Student’s t-test.

Because neuronal MNs formed during development can be incorporated into microglia and alter microglial properties (Yano et al., 2025), we investigated whether MNs arising during aging are also incorporated into microglia. First, we examined whether MNs localized to non-neuronal spaces in the aged brain and found that MNs were present in MAP2-negative (MAP2^−^) regions (Figure 3A). We next examined whether these MNs were taken up by astrocytes; however, they were rarely engulfed. By contrast, a subset of MNs was incorporated into microglia, although population is not high (Figures 3B–F). These results suggest that neuronal MNs are released from neurons and preferentially incorporated into microglia, but not efficiently into astrocytes. To test whether MN incorporation affects microglial characteristics, we analyzed microglial morphology. In contrast to postnatal stages, MN^+^ microglia exhibited elongated processes and an increased number of cellular processes (Figures 3G–I). Additionally, this effect was attenuated by administration of the P2Y6 receptor antagonist MRS2578, which suppresses microglial engulfment (Koizumi et al., 2007) (Figures 3J–L). These data suggest that neuronal MNs incorporated into microglia induce microglial morphological remodeling in aged microglia, although their properties may differ from those observed during development.

### MN propagation alters a hallmark of aged microglia

**Figure 4:**
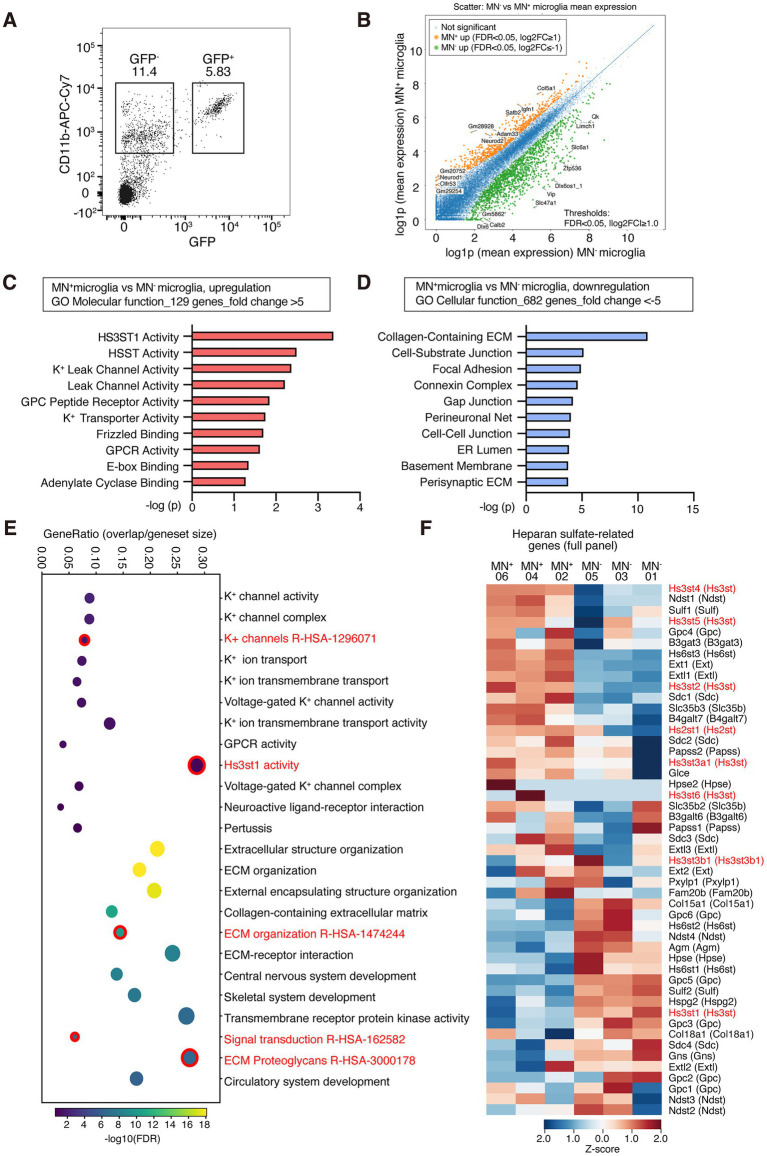
MN propagation alters a hallmark of aged microglia. **(A)** Representative gating strategy for flow cytometry of either GFP^+^ or GFP^−^ microglia from the NexCre: LSL-Sun1GFP whole brain (20, 20, and 25 months of age, 3 brains). Numbers above the gate, percentage of total cell count. **(B)** Scatter plot indicating the ratio and differences of FPKM between GFP^+^ microglia and GFP^−^ microglia. FDR < 0.05, log2FC > 1.0, Extract the top 10 genes for each set of differentially expressed genes (DEGs). **(C,D)** GO enrichment analysis of DEGs. Data were analyzed using the Enrichr analysis tool. **(E)** Pathway enrichment analysis was performed to compare the upregulated genes between GFP^+^ microglia and GFP^−^ microglia. Dot size represents GeneRatio and color indicates −log10(FDR); pathways related to heparan sulfate (HS) were highlighted with red outlines based on HS-related keywords in pathway names. **(F)** Heatmap comparing FPKM between GFP^+^ microglia and GFP^−^ microglia. Red; heparan sulfate glucosamine sulfotransferase family. HS-related genes were extracted from the previous studies (Bishop et al., 2007; Iozzo et al., 2009).

Given that MNs appeared to affect microglial states in the developing brain, we next examined whether MN incorporation also alters microglial gene expression during aging. CD11b^+^ GFP^+^ and CD11b^+^ GFP^−^ microglia were isolated from the brains of aged NexCre; LSL-Sun1-GFP mice (Figure 4A) and subjected to bulk RNA-sequencing (RNA-seq), followed by gene set enrichment analysis (Figure 4B). Accordingly, microglia incorporating MN (GFP^+^ microglia) displayed increased expression of heparan sulfate glucosamine 3-O-sulfotransferase (*Hs3st4*, *Hs3st5*, *Hs3st2*, *Hs3st3a1*, *Hs3st6*) and members of the Exostosin, which regulate HS chain elongation (*Ext1*, *Extl1*, *Extl3*, *Ext2*), compared with GFP^−^ microglia. By contrast, GFP^+^ microglia displayed decreased expression of bifunctional heparan sulfate N-deacetylase/N-sulfotransferase (*Ndst2*, *Ndst3*, *Ndst4*) and Glypican family proteins (*Gpc1*, *Gpc2*, *Gpc3*, *Gpc5*, *Gpc6*) (Figures 4C–F). These results suggest that neuronal MNs formed during aging reshape microglial gene expression programs associated with HS synthesis pathways.

## Discussion

**Figure 5:**
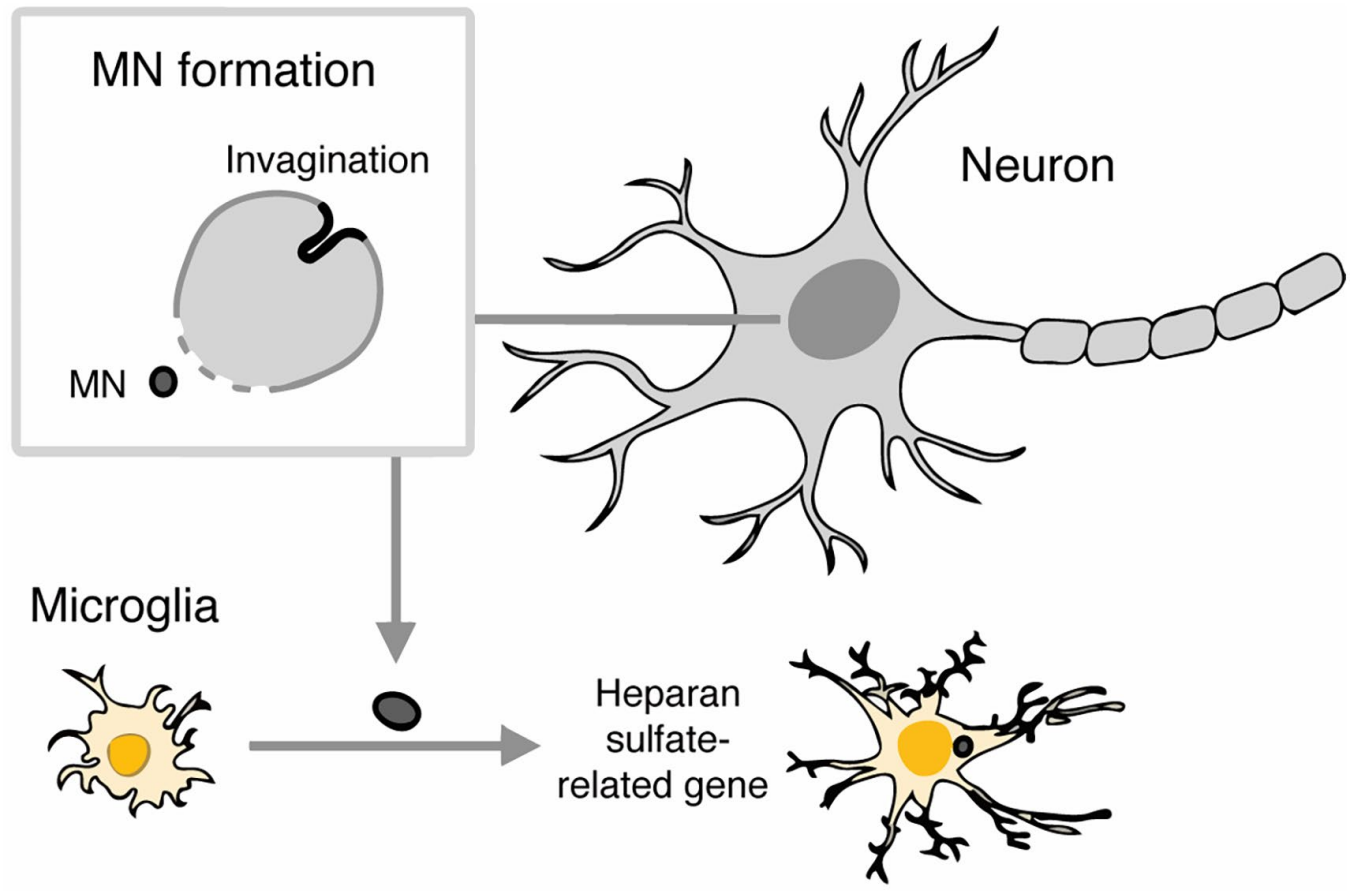
Model for the MN transfer from aged neurons to microglia. Age-related nuclear envelope fragility leads to the generation of MN, which are transferred to microglia and changes in the expression of HSPG-related genes.

In this study, we demonstrate the following findings. First, aging neurons exhibit increased nuclear envelope fragility, leading to the generation of MNs. Second, microglia in the aged brain incorporate neuronal MNs. Third, microglia incorporating micronuclei exhibit distinct gene expression patterns (Figure 5). Although these phenomena share similarities with MN propagation observed during brain development (Yano et al., 2025), we also identify several differences, including the sets of expression genes in microglia and the morphological features of MN^+^ microglia. These distinctions suggest that MN propagation in the aged brain may represent a biologically distinct process from that observed during development.

We found that MN formation in aged neurons is associated with nuclear envelope invagination, which was in turn linked to aberrant microtubule localization. However, the mechanisms linking invagination to MN formation have yet to be fully elucidated. It has been shown that the physiological stresses such as blood–brain barrier disruption and vascular leakage are known to affect surrounding neurons. Such factors could disrupt neuronal cytoskeletal organization, potentially leading to nuclear envelope deformation and MN formation. Indeed, our data show that MNs are frequently observed in deep cortical layers, where age-associated vascular damage is particularly prominent. Additionally, our preliminary data show that treatment of primary cultured neurons with serum from aged mice increase in nuclear envelope invagination. Thus, MN formation in aged neurons may reflect not only neuron-intrinsic alterations but also age-related changes within the neurovascular unit. A key question is why nuclear envelope invaginations are associated with MN formation. One potential mechianism is that nuclear envelope invagination imposes mechanical stress across the nucleus, generating increased stress on the opposing side and thereby creating a cellular environment that favors MN formation. Previous studies have shown that membrane damage is recognized by the endosomal sorting complexes required for transport (ESCRT) complexes, which mediate the removal of damaged membrane regions (Jimenez et al., 2014). Consistent with this idea, we observed that aged neuronal MNs primarily contain nuclear membrane components and relatively little chromatin. These observations raise the possibility that ESCRT complexes are recruited to regions destined to form MN and selectively excise segments of the damaged site, thereby giving rise to MN-like structures.

It has been shown that the expression levels of Lamin A and Lamin B1 are reduced in the aged brain (Scaffidi and Misteli, 2006; Freund et al., 2012). Since these proteins are components of the nuclear lamina and act as an anchor of SUN1, changes in their expression levels may affect the integrity of the nuclear envelope. Because SUN1-GFP expression in these mice is induced by Nex-Cre–mediated recombination of a loxP-stop-loxP-SUN1-GFP allele, it may be possible that reduced Lamin and increased SUN1-GFP perturb nuclear membrane integrity. However, thus far, we have not detected any significant difference in MN formation between wild-type and SUN1-GFP mice. Therefore, we speculate that other factors, such as aged serum, may act as triggers for MN formation.

Previously, we found that neuronal MNs are released as vesicular structures and subsequently taken up by microglia during the postnatal stage, inducing a reduction in microglial complexity (Yano et al., 2025). In contrast, MNs observed in aged neurons differ in both morphology and properties from those present during development. Because the mechanisms underlying MN production and propagation differ between the developmental and aged stages, their roles and physiological significance may also differ as a consequence. So far, we have observed a trogocytosis-like phenomenon in which a subset of microglia contacts and prune portions of neuronal structures. This observation implies that microglia may directly acquire MNs from aged neurons. Microglia express multiple phagocytosis-related receptors, including TREM2 (Bosco et al., 2025). Neurons undergoing age-associated cell death may generate apoptotic bodies, which could be internalized by microglial phagocytic receptors. Indeed, we found that the P2Y6 receptor antagonist, which suppresses MN incorporation, attenuates MNs-dependent morphological changes in aged brain. Therefore, it is plausible that MN propagation in the aged brain may occur through mechanisms that differ from those in the developing brain.

It has been reported that DNA damage in the aged microglia causes DNA to leak into the cytoplasm. This leaked DNA is subsequently encapsulated into EVs and released into the extracellular space, thereby inducing neuroinflammation (Arvanitaki et al., 2024). These findings suggest a potential mechanism of EV-mediated intercellular communication between neurons and microglia. In contrast, our RNA-seq analysis revealed that microglia that had internalized MNs exhibited a global decrease in the mRNA levels of exosome marker genes (*Cd9*, *Cd63*, and *Cd81*). This suggests that microglia that have taken up MNs are unlikely to subsequently secrete EVs that affect neurons. Notably, CD9 in neural stem cells has been suggested to rejuvenate surrounding cells via exosome secretion (Sun et al., 2025). This raises the possibility that MN propagation affects not only neurons and microglia but also neighboring cells, thereby regulating the microenvironmental conditions.

Despite our observation that neuronal MNs alter microglial gene expression during aging, the signaling pathways responsible for MN incorporation-related induction of HS genes remain unclear. Previous studies have reported that neuronal MNs formed during development contain genomic DNA and regulate the cGAS function in microglia (Yano et al., 2025), implying that the cGAS-STING axis may function as a regulatory pathway promoting heparan sulfate proteoglycan (HSPG) synthesis. On the other hand, MNs derived from aged neurons may exhibit weak DAPI staining signals, suggesting reduced chromatin content or even a structure composed solely of the nuclear envelope. In such cases, these MNs may modulate intracellular signaling in microglia through mechanisms other than cGAS. A plausible signaling pathway to consider is lysosomal stress. Previously, we reported that autophagic pathway plays an important role in regulating MN secretion (Asami et al., 2025). It is also known that excessive phagocytic activity imposes a burden on the lysosome of microglia, leading to activation of the lysosomal stress response and the lysosomal stress sensor, TFEB (Medina et al., 2015). As aged microglia are required to clear substantial amounts of dead cells and waste products, the uptake of MNs may impose a burden on lysosomal function. In relation to these findings, it cannot be ruled out that MN propagation does not directly regulate gene expression in microglia; rather, microglia with altered properties may become more prone to taking up MN. In postnatal microglia, *in vivo* imaging using two-photon excitation microscopy has revealed that their processes become shorter and the cells migrate toward the brain surface after incorporating MN. Therefore, we are considering not only the possibility that aged MN act as mediators that induce microglial transformation, but also the possibility that they are processed as cellular waste at least aged brain. Indeed, we have found that MN^+^ microglia extend their processes toward blood vessels and preferentially contact vulnerable vasculature. These observations raise the possibility that microglia may facilitate the clearance of MN recognized as cellular waste.

Microglia incorporating MNs exhibit altered gene expression in the HSPG biosynthesis pathway. Notably, the expression of glypican, a representative core protein of HSPG, was decreased, whereas the expression of HS-modifying enzymes was increased. HSPGs have been reported to act as damage-associated molecular patterns (DAMPs) that activate receptors such as TREM2 and TLRs (Chan et al., 2020). In addition, HS regulates CD14-dependent TLR4 signaling in microglia and promote inflammatory cytokine production (O'Callaghan et al., 2015). Therefore, the MN-associated upregulation of HSPG expression may contribute to enhanced inflammatory responses in the surrounding tissue. On the other hand, HSPG functions to trap morphogens like Wnt (Baeg et al., 2004) and growth factors, thereby enhancing intracellular signaling. Recent studies have also shown that HSPGs can sequester VEGF, modulate the vascular microenvironment, and contribute to post-stroke recovery (Chan et al., 2020). Based on these findings, we hypothesize that microglia recognize compromised vasculature and promote a switch in HSPG core proteins along with increased HS production, thereby supporting vascular function during aging.

Overall, we demonstrate that microglia incorporate neuronal MNs even during aging. However, MNs present in aged neurons are likely to differ from those observed during development in both morphology and properties. We found that microglia incorporating MNs exhibit altered expression of genes involved in HSPG pathways. On the other hand, there are still several experimental limitations and unresolved questions in this study. First, since this study used aged SUN1-GFP mice, the sample size was limited. In addition, the propagation of MNs in aged mice may vary among individuals; therefore, future studies with larger sample sizes will be important. Second, it remains unresolved whether MNs actively function as signaling entities that induce these transcriptional changes, or whether microglia expressing specific HSPG-related genes preferentially incorporate MNs. Lastly, we cannot exclude the possibility that these MNs are recognized and eliminated as cellular debris rather than acting as functional signals in the aged brain. Although overcoming these issues are still challenging, recent progress has been made in the development of AAVs capable of gene delivery into microglia. By optimizing a system for AAV delivery, it may become possible to overcome the weaknesses and experimental limitations. Thus, further studies are expected to clarify the physiological significance of MN propagation in the aged brain.

## Data Availability

Sequence data have been deposited in the DNA Data Bank of Japan (DDBJ) Sequence Read Archive under the accession code DRA026188, https://ddbj.nig.ac.jp/search/entry/sra-submission/DRA026188.

## Footnotes

1
https://maayanlab.cloud/Enrichr/
